# Utility of clinical parameters to identify HIV infection in infants below ten weeks of age in South Africa: a prospective cohort study

**DOI:** 10.1186/1471-2431-11-104

**Published:** 2011-11-21

**Authors:** Heather B Jaspan, Landon Myer, Shabir A Madhi, Avy Violari, Diana M Gibb, Wendy S Stevens, Els Dobbels, Mark F Cotton

**Affiliations:** 1 Children's Infectious Diseases Clinical Research Unit, Department of Paediatrics and Child Health and Centre for Infectious Diseases, Faculty of Health Sciences, Stellenbosch University, Tygerberg, South Africa; 2 School of Child and Adolescent Health, University of Cape Town, Cape Town, South Africa; 3 Children's Hospital and Regional Medical Centre, Department of Pediatrics, University of Washington, Seattle, WA, USA; 4 Infectious Diseases Epidemiology Unit, School of Public Health & Family Medicine, University of Cape Town, Cape Town, South Africa & Department of Epidemiology, Mailman School of Public Health, Columbia University, USA; 5 Department of Science and Technology/National Research Foundation: Vaccine Preventable Diseases; & MRC/WITS Respiratory and Meningeal Pathogens Research Unit; University of the Witwatersrand, Johannesburg, South Africa; 6 Perinatal HIV research Unit, University of the Witwatersrand, Johannesburg, South Africa; 7 Medical Research Council, Clinical Trials Unit, London. UK; 8 Department of Molecular Medicine and Haematology, University of the Witwatersrand and National Health Laboratory Service, Johannesburg, South Africa; 9 Seattle Biomedical Research Institute, 307 Westlake Ave S, Seattle WA 98109 or University of Cape Town Health Sciences Faculty, Anzio Rd Observatory, Cape Town 7925, South Africa

## Abstract

**Background:**

As HIV-infected infants have high mortality, the World Health Organization now recommends initiating antiretroviral therapy as early as possible in the first year of life. However, in many settings, laboratory diagnosis of HIV in infants is not readily available. We aimed to develop a clinical algorithm for HIV presumptive diagnosis in infants < 10 weeks old using screening data from the Children with HIV Early Antiretroviral therapy (CHER) study in South Africa.

HIV-infected and HIV-uninfected exposed infants < 10 weeks of age were identified through Vertical Transmission Prevention programs. Clinical and laboratory data were systematically recorded, groups were compared using Kruskal-Wallis, analysis of variance (ANOVA), and Fisher's exact tests. Receiver Operating Characteristic (ROC) curves were compiled using combinations of clinical findings.

**Results:**

417 HIV-infected and 125 HIV-exposed, uninfected infants, median age 46 days (IQR 38-55), were included. The median CD4 percentage in HIV-infected infants was 34 (IQR 28-41)%. HIV-infected infants had lower weight-for-age, more lymphadenopathy, oral thrush, and hepatomegaly than exposed uninfected infants (Adjusted Odds Ratio 0.51, 8.8, 5.6 and 23.5 respectively; p < 0.001 for all). Sensitivity of individual signs was low (< 20%) but specificity high (98-100%). If any one of oral thrush, hepatomegaly, splenomegaly, lymphadenopathy, diaper dermatitis, weight < 50^th ^centile are present, sensitivity for HIV infection amongst HIV-exposed infants was 86%. These algorithms performed similarly when used to predict severe immune suppression.

**Conclusions:**

A combination of physical findings is helpful in identifying infants most likely to be HIV-infected. This may inform management algorithms and provide guidance for focused laboratory testing in some settings, and should be further validated in these settings and elsewhere.

## Background

Identifying HIV infection in early infancy is an important challenge to effective paediatric HIV care and treatment. HIV-infected infants have a high mortality, particularly in heavily disease-burdened settings [1-6]. The Children with HIV Early Antiretroviral Therapy (CHER) trial [4] recently showed that early antiretroviral therapy (ART), commenced at a median of 7 weeks of age was associated with a 75% reduction in mortality versus the prevailing standard of care at the time (the WHO 2006 guidelines) [7]. Importantly, most deaths occurred in the first few months of life and none of the infants had a CD4 < 25% or advanced HIV disease according to Centers for Diseases Control criteria [4].

Diagnostic HIV-1 DNA PCR is recommended at 6 weeks of age in HIV-exposed infants going through Vertical Transmission Prevention (VTP) programs [8]. Although qualitative HIV-1 PCR testing is becoming more available, turn-around time for results and subsequent initiation of ART can take weeks. Clinical algorithms for HIV diagnosis in young infants could be useful for identifying those with probable HIV infection [9-13]. Interventions might be implemented for fast-tracking early diagnosis.

Clinical algorithms for pediatric HIV diagnosis [9-13] have been developed but very few have used data on clinical manifestations from very young infants. To our knowledge, only one algorithm for this age group has been developed in Zimbabwe [9], using data from the pre-VTP era. Our aim was to investigate the predictive value of clinical characteristics for HIV infection in HIV-infected and HIV-exposed uninfected infants and to develop a clinical algorithm for identification of HIV infection in young infants who have failed VTP interventions.

## Methods

### Participants, data collection and laboratory methods

Data were collected during the screening phase of two clinical trials between 2005 to 2007 in Cape Town and Soweto, South Africa; the CHER trial, and a parallel observational cohort study. For randomization in the CHER study, baseline CD4 had to be ≥ 25% [4]. A small number of infants with lower CD4 percentage were enrolled in the parallel observation cohort, so were included in the present analysis. Infants were identified through VTP and referred to the study sites. VTP consisted of single dose nevirapine (NVP) to mother and newborn; and in Cape Town, Zidovudine (ZDV) was given to mothers from 32 weeks gestation, and to the infant for one week. HIV-exposed infants were tested by HIV-1 DNA PCR (Roche Amplicor HIV-1 DNA assay version 1.5, Roche Molecular Systems, Inc., Branchburg, NJ) between 4 and 10 weeks of age. At screening, clinical signs were recorded and HIV infection was confirmed by plasma HIV RNA level > 5000 copies per ml (Roche Ampliprep/Cobas Amplicor assay, Roche Molecular Systems, Inc., Branchburg, NJ) (see below). High viral loads were not titrated beyond 750,000 viral copies/mL (log 5.88). HIV-exposed infants testing HIV-1 DNA PCR negative were still eligible for the cohort study, and were included in this analysis. HIV-infected infants had lymphocyte subsets measured using Beckman Coulter single platform lyse-no wash procedure using Immunoprep™ reagents with Flow Count™ fluorospheres. A small number of HIV-infected infants with CD4% below 25% were eligible for the concurrent cohort study and were therefore included in the analysis.

Data from screening for both studies were collected on predefined questionnaires by doctors aware of the infants' HIV status, but unaware of their degree of immunosupression. Reflux was defined as excessive vomiting after feeds. Hepatomegaly was defined as a palpable liver > 1 cm below the costal margin in the right mid-clavicular line. Splenomegaly was noted if the spleen was palpable. Encephalopathy was defined as symmetrically abnormal neurological examination, microcephaly or lack of head circumference growth, or cerebral atrophy on CT scan. Lymphadenopathy was defined as palpable lymph nodes greater than 1 cm in diameter. Lymphadenopathy was generalised if it was palpable in two or more areas including cervical, axillary, or inguinal regions. Diaper dermatitis was an abnormal rash of any type in the diaper area, candidal or otherwise. Standard World Health Organisation growth curves (available at http://www.who.int/childgrowth/standards/en/) were used to define cut-off values for 3^rd^, 15^th^, and 50^th ^centiles (marked on the charts) for weight-for-age. Anemia and neutropenia were defined according to the Division of AIDS toxicity table for grading severity of Pediatric (≤ 3 months of age) adverse experiences (available at http://rsc.tech-res.com/safetyandpharmacovigilance/gradingtables.aspx)

### Statistical methods

Stata version 9.0 (College Station, Texas) was used for analysis. Screening data were compared using Kruskal-Wallis tests, analysis of variance (ANOVA), and Fisher's exact tests. Similar analysis was performed for CD4% data. Multivariate regression was performed including age as a dependent variable to determine the relationship between log viral load and clinical findings. We estimated sensitivity, specificity, positive likelihood ratios, and age-adjusted odds ratios (OR), for the association between HIV status and clinical parameters. The 95% confidence intervals (CI) were calculated using standard methods. Receiver Operating Characteristic (ROC) curves were examined using combinations of clinical predictors of HIV infection. Characteristics with significant age-adjusted OR in univariate analyses (p < 0.05) were combined to achieve the highest Area Under the Curve (AUC) for the ROC.

### Ethical Considerations

The Ethics Committees of the Universities of Stellenbosch and Witwatersrand approved the studies. Written informed consent was obtained from legal guardians of all infants.

## Results

417 HIV-infected and 125 HIV-exposed, uninfected (HEU) infants were included. The median age at screening was 46 days (Interquartile range, [IQR] 38-55). The median CD4% of the HIV-infected infants was 34% (IQR 28-41). Sixty-two HIV-infected infants with CD4 < 25% were included in the analysis.

Only 12% of infants were ever breastfed; uptake of VTP was 90%. The demographics and prevalence of selected clinical characteristics of infants by infection status at screening are shown in Table 1. Weight-for-age of HIV-infected infants was significantly lower than in HIV-exposed uninfected infants. Infected infants were more likely to have weight below the 3^rd ^and 15^th ^but not the 50^th ^centile. Lymphadenopathy (axillary, cervical, generalised, or any), oral thrush, splenomegaly, or hepatomegaly and a history of reflux were more common in infected infants. Diaper dermatitis (candidal, atopic, or other) was also significantly associated with HIV status. Both anemia and neutropenia of grade 2 or above were almost exclusively found in HIV-infected infants. Electrolyte and liver function test results did not differ between the groups (data not shown). However, 27% of HIV-infected infants had weight above the 50^th ^centile.

**Table 1:** Frequency of selected physical findings and clinical events in very young HIV-infected and exposed uninfected infants.

	Exposed uninfected n = 125	Infected n = 417	**Unadjusted p-value**^**3**^	**Adjusted Odds Ratio (95% CI)**^**3**^
**Female gender (%)** **Median age, days (IQR)**	57 (46) 48 (38-51)	239 (57) 45 (38-56)	0.18 0.07^1^	
***Anthropometry at screening***				
**Median weight, kg (IQR)**	4.8 (4.3-5.2)	4.4 (3.9-4.9)	< 0.001^2^	0.51 (0.38-0.68)
**Weight < 3^rd ^centile (%)**	8 (6.4)	79 (18.9)	< 0.001	3.2 (1.4-6.8)
**Weight < 15^th ^centile (%)**	23 (18.4)	166 (39.8)	< 0.001	2.8 (1.7-4.6)
**Weight < 50^th ^centile (%)**	79 (63.2)	303 (72.7)	0.04	1.5 (0.98-2.3)
**Median length, cm**	55 (53-56)	54 (52-56)	0.78^2^	0.97 (0.91-1.04)
**Median HC, cm**	39 (38.4-40)	39 (38-40)	0.20^2^	0.95 (0.88-1.03)
***Physical exam findings at screening***				
**Any LAD^# ^(%)**	6 (5)	132 (32)	< 0.001	8.8 (3.8-20.9)
**Candidal rash (%)**	8 (6)	46 (11)	0.17	1.6 (0.8-3.6)
**Atopic dermatitis (%)**	13 (10)	83 (20)	0.01	2.0 (1.1-3.7)
**Diaper dermatitis**	23 (18)	149 (36)	< 0.001	2.4 (1.5-4.0)
**Oral thrush (%)**	13 (10)	163 (39)	< 0.001	5.6 (3.0-10.2)
**Hepatomegaly (%)**	1 (1)	70 (17)	< 0.001	23.5 (3.2-170.8)
**Splenomegaly (%)**	0 (0)	30 (7)	< 0.001	*
***Clinical event prior to or at screening***				
**Gastroenteritis (%)**	8 (6)	44 (11)	0.17	1.7 (0.8-3.7)
**Otitis media (%)**	4 (3)	6 (1)	0.20	0.4 (0.1-1.5)
**Sepsis (%)**	3 (2)	12 (3)	1.00	1.0 (0.3-3.7)
**URTI (%)**	24 (19)	81 (19)	1.00	1.0 (0.6-1.7)
**Reflux (%)**	0 (0)	15 (4)	**0.03**	*
**Pneumonia (%)**	7 (6)	29 (7)	0.67	1.2 (0.5-2.8)
***Laboratory findings***				
**Anemia (%)**	0 (0)	22 (5)	**0.004**	*
**Neutropenia (%)**	0 (0)	15 (4)	**0.03**	*

^1 ^Kruskal Wallis ^2^ANOVA ^3^Fishers exact unless otherwise specified

^3^Age-adjusted OR of infected infants versus HIV exposed-uninfected infants

IQR - Interquartile range. CI - Confidence Interval. HC - Head circumference. URTI - Upper respiratory tract infection.

#Any lymphadenopathy - includes generalized, cervical, axillary or inguinal

*Small sample sizes prevented the calculation of adjusted OR

The 62 HIV-infected infants with CD4% < 25 had a median age of 51.5 days [IQR 42-66]. Their median CD4% was 20.9 [IQR 17.6-23.3]. Compared with those with CD4 ≥ 25%, severely immunocompromised infants were older and more likely to have weight below 3^rd ^and 15^th ^centile for age, but not 50^th ^(Table 2). Severely immunosuppressed infants were also more likely to have lymphadenopathy, hepatomegaly, splenomegaly, and a history of gastroenteritis or pneumonia.

**Table 2:** Frequency of selected physical findings and clinical events in very young HIV-infected with CD4% < 25% (severe immune suppression) versus CD4 ≥ 25%

	CD4 < 25% n = 62	CD4 ≥ 25% n = 313	**Unadjusted p-value**^**3**^	**AOR (95% CI)**^**3**^
**Female gender (%)**	29 (46.8)	187 (59.7)	0.06	
**Median age, days (IQR)**	52 (42-66)	45 (39-55)	0.002^1^	
***Anthropometry at screening***				
**Median weight, kg (IQR)**	4.3 (3.9-5)	4.4 (4.0-4.9)	0.228	1.6 (1.1-2.4)
**Weight < 3^rd ^centile (%)**	19 (30.6)	52 (16.6)	0.010	NA
**Weight < 15^th ^centile (%)**	36 (56.4)	115 (37.0)	0.004	NA
**Weight < 50^th ^centile (%)**	51 (82)	228 (72.8)	0.121	NA
**Median length, cm**	54 (52.5-55)	54 (52-56)	0.563	1.1 (0.98-1.2)
**Median HC, cm**	39 (38-40)	39 (38-40)	0.859	1.1 (0.9-1.1)
***Physical exam findings at screening***				
**Any LAD^# ^(%)**	33 (53.2)	93 (29.7)	< 0.001	0.4 (0.2-0.7)
**Atopic dermatitis (%)**	14 (22.6)	64 (20.4)	0.705	1.1 (0.5-2.1)
**Diaper dermatitis**	28 (45.2)	111 (35.5)	0.149	0.7 (0.4-1.2)
**Oral thrush (%)**	29 (46.8)	115 (36.7)	0.138	0.7 (0.4-1.1)
**Hepatomegaly (%)**	11 (17.7)	19 (6.1)	0.002	0.4 (0.2-0.8)
**Splenomegaly (%)**	19 (30.6)	51 (16.3)	0.008	0.5 (0.3-0.9)
***Clinical event prior to or at screening***				
**Gastroenteritis (%)**	12 (19.4)	29 (9.3)	0.020	0.5 (0.2-1.0)
**Otitis media (%)**	1 (1.6)	5 (1.6)	0.993	1.5 (0.2-13.8)
**Sepsis (%)**	4 (6.5)	7 (2.2)	0.072	0.4 (0.1-1.5)
**URTI (%)**	11 (17.7)	65 (20.8)	0.558	1.2 (0.6-2.5)
**Reflux (%)**	3 (4.8)	11 (35.1)	0.615	0.6 (0.2-2.4)
**Pneumonia (%)**	11 (17.2)	17 (5.4)	0.001	0.2 (0.1-0.6)
***Laboratory findings***				
**Anemia (%)**	4 (6.5)	18 (5.8)	0.830	1.0 (0.3-3.0)
**Neutropenia (%)**	4 (6.5)	9 (2.9)	0.160	0.4 (0.1-1.2)

^1 ^Kruskal Wallis ^2^ANOVA ^3^Fishers exact unless otherwise specified

^3^Age-adjusted OR of HIV-infected infants with CD4 ≥ 25% versus those with CD4 < 24%

IQR - Interquartile range. CI - Confidence Interval. HC - Head circumference. URTI - Upper respiratory tract infection.

#Any lymphadenopathy - includes generalized, cervical, axillary or inguinal

*Small sample sizes prevented the calculation of adjusted OR

The median log viral load of the HIV-infected infants was 5.88 [Range 3.78-5.88]. In a univariate regression model, there was weak association between log viral load and age (regression coefficient 2.77; 95% CI -0.53 - 6.06). No age-adjusted clinical finding or event was predictive of higher viral load except oral thrush (regression coefficient 0.09, 95% CI 0.02; 0.16).

The sensitivity, specificity, and positive likelihood ratio (LR+) of selected findings for HIV infection is shown in Table 3. Low weight-for-age, any lymphadenopathy, hepatomegaly, splenomegaly, oral thrush, and reflux were all highly specific but insensitive for HIV infection. Excluding infants with weight above the 50^th ^centile, as in the algorithm by Iliff et al [9] improved sensitivity and specificity.

A combination of various clinical findings in a ROC improved sensitivity without compromising specificity (Table 4). For example, if any one of six clinical findings - oral thrush, hepatomegaly, splenomegaly, lymphadenopathy, diaper dermatitis, or weight below the 15^th ^centile were present, the sensitivity for HIV infection amongst HIV-exposed infants was 78% and the specificity 62%. Increasing the weight cut-off to < 50^th ^centile improved sensitivity to 89% (Table 4). By excluding diaper dermatitis, including reflux, and changing the weight cut-off to below the 3^rd ^centile, specificity increased to 80% but sensitivity decreased to 64%. When cut-offs to any two findings were increased, specificity approached 100%, but compromised sensitivity. Although nurses can be trained to recognise hepatosplenomegaly and lymphadenopathy, simple algorithms may be preferred by programs with high-volume or nurse-run programs. Table 4 shows simplified algorithms including only diaper dermatitis, oral thrush, and weight < 3^rd ^and 50^th ^centiles, giving sensitivities ranging from 61 to as high as 85%, with that for 15^th ^centile falling somewhere in between (data not shown).

**Table 3:** Sensitivity and specificity of select clinical findings and events for distinguishing HIV-infected from exposed uninfected infants

	All exposed infants		
Characteristic	Sensitivity (95%CI)	Specificity (95%CI)	+ LR
General LAD	14.6 (11.8-17.9)	97.6 (93.1-99.5)	6.1
Any LAD	24.4 (20.9-28.3)	95.2 (89.8-98.2)	4.8
Hepatomegaly	13.0 (10.2-16.1)	99.2 (95.6-100)	13
Splenomegaly	5.6 (3.8-7.8)	100	n/a
Oral thrush	30.2 (26.3-34.2)	89.6 (82.8-94.3)	164
Diaper rash	35.7 (31.1-40.5)	81.6 (12-26.3)	1.9
Reflux	2.8 (1.6-2.5)	100	n/a
Weight < 3^rd^	14.6 (11.7-17.9)	93.6 (87.8-97.2)	2.3
Weight < 15^th^	30.7 (26.9-34.8)	81.6 (73.7-87.9)	1.7

WAZ - weight-for-age score. CI - confidence interval.

+LR - positive likelihood ratio. n/a - not applicable.

When these algorithms were applied to HIV-infected infants to predict severe immune suppression (CD4 < 25%), the algorithms were sensitive, but poorly specific using one cut-off finding. For example, any one of the six clinical findings in Table 4B (weight below 50^th ^centile, hepatomegaly, splenomegaly, diaper dermatitis, and lymphadenopathy) had a sensitivity of 89% but specificity of 2% for predicting severe immune suppression.

**Table 4:** Receiver Operating Characteristic (ROC) curves of combinations of clinical findings with the greatest area under the curve (AUC), and sensitivity and specificity of various cut-off numbers of findings in predicting HIV-infection among exposed infants.

One of a combination of oral thrush, any lymphadenopathy, hepatomegaly, splenomegaly, plus:	ROC AUC	Findings present	Sensitivity (%)	Specificity (%)
A: weight < 15th centile, diaper dermatitis	0.754	1	77.9	61.2
		2	47.7	88.0
		3	25.2	98.4
B: weight < 50th centile, diaper dermatitis	0.717	1	88.9	29.6
		2	48	90.4
		3	20.6	99.2
C: weight < 3rd centile, reflux	0.744	1	63.8	80
		2	31.4	97.6
		3	12.5	100

**One of a combination of oral thrush, diaper dermatitis, plus:**	**ROC AUC**	**Findings present**	**Sensitivity (%)**	**Specificity (%)**
D: weight < 3^rd ^centile	0.682	1	60.7	72
		2	28.3	92.8
		3	4.8	100
E: weight < 50^th ^centile	0.666	1	84.7	30.4
		2	46.8	82.4

## Discussion

We found that certain clinical signs and symptoms are highly specific for HIV infection in relatively healthy infants exposed to ARVs for prevention of vertical transmission below 10 weeks of age. Any one of a complex of findings including oral thrush, lymphadenopathy, hepatomegaly, splenomegaly, weight centile, diaper dermatitis, or reflux, give specificities and sensitivities of up to 89% and 80% respectively. A small number of studies prospectively investigated clinical symptoms in acutely infected infants during breastfeeding. In a study by Rouet et al in Cote d'Ivoire, 22 of 342 infants developed postnatal HIV infection. Acute retroviral syndrome, generalised lymphadenopathy and dermatitis were predictive of acute HIV infection [14]. Richardson et al performed a similar study in Kenya where 40 (71%) of acutely infected infants were below 2 months of age in a cohort of 189 infants. Acute infection was associated with lymphadenopathy in ≥ 2 sites and upper respiratory tract infection [15].

In a larger study from the pre-VTP era of infants born to HIV-infected women, the Zimbabwe Vitamin A for Mothers and Babies (ZVITAMBO) group evaluated WHO, South African, and Zimbabwean algorithms for HIV diagnosis in 6-week-old infants [9]. In this study, 52% of HIV-infected infants had a weight for age < 50^th ^centile (Z score ≤ 0), and by focussing on this group, the author's developed diagnostic algorithms based on clinical signs with relatively high yield. However, by focussing only on HIV-exposed infants with weight-for-age below the median, sensitivity of their clinical algorithm for HIV diagnosis was 65%. When combined with ≥ two other physical signs (e.g. pneumonia, ear discharge, treatment for TB, oral candidiasis), the sensitivity decreased to 20% but the specificity increased to 94%. Our algorithm including weight < 50^th ^centile (algorithm B; table 3) but with different findings improved sensitivity to 86% in this age group. Additionally, a simplified algorithm that included weight < 50^th ^maintained high sensitivity of 85%. However, algorithms of this sort exclude the high proportion of HIV-infected infants with weight above 50^th ^centile.

The ZVITAMBO approach [9] of focusing where yield for subsequent DNA PCR testing may be highest appears useful in settings with severe resource limitations [16]. South Africa is a mid-income country that can afford HIV-1 diagnostic PCRs on all HIV-exposed infants. Therefore, although limiting tests to infants below the 50^th ^centile may reduce costs, the number of infants missed will be unacceptably high. Consequently, an algorithm with higher sensitivity would be useful. Additionally, in settings where PCR is unavailable or turnaround time for results unacceptable, an algorithm with high sensitivity may be preferred as a strategy for ART initiation to prevent the early infant mortality associated with HIV infection. Therefore using algorithm B, may be preferable in these settings. The advantage of algorithms such as ours is that programs could adapt the cut-off to suit their needs.

To our knowledge, ours is the first study to investigate diagnostic algorithms of predominantly formula fed infants in the VTP era. Our algorithms have the highest sensitivity for HIV identification of all those previously published, particularly in this age group.

The majority of other algorithms and guidelines to identify HIV-infected children have not included infants below two months of age [10-12]. Jones et al found a sensitivity of 17% when applying the Integrated Management of Childhood Illness (IMCI) algorithms for predicting pediatric HIV infection to a cohort of 301 HIV-exposed infants in VTP programs, of whom 26 (8.7%) were shown to be HIV-infected by HIV DNA PCR [10]. However, using clinical signs similar to those selected in our study (hepatosplenomegaly, recurrent infections, failure to thrive, and candidiasis) and where doctors evaluating the infants were unaware of infection status, the sensitivity for HIV infection increased to 56% at 6 weeks of age [10].

As expected, increasing the number of positive signs reduces sensitivity but increases specificity. Reflux occurred at low frequency (3.4%) but appeared very specific, albeit difficult to define. Of note, 11 (73%) of the 15 infants with reflux also had oral thrush, which could have aggravated or caused the symptom; swallowing incoordination, an early sign of encephalopathy, could also have contributed. That oral thrush and low weight for age were so highly specific in this population suggests that these findings cannot only be attributed to formula feeding or co-morbidities such as malnutrition and TB.

A limitation of our study is that clinician's knowledge of the infants' HIV status may have influenced the finding and reporting of clinical findings. On the other hand, HIV-infected infants eligible for CHER were selected for lack of severe clinical disease [4] and doctors trained for the study evaluated all infants. Our results require validation in other settings, for example, where malaria and breastfeeding are prevalent, and in VTP programs with larger numbers of HIV-exposed uninfected infants and with lower HIV prevalence. Our study included a large group of HIV-infected infants (77%) and a smaller number of HIV-exposed infants, all below 10 weeks of age, in contrast to all of the other

studies. Also, as mentioned, infants with severe clinical disease, where the diagnosis may be more obvious, were also excluded. In settings where this algorithm would be useful, the prevalence of HIV-infection amongst exposed infants would be lower, and thus affect the positive and negative predictive values of the algorithm. In addition, due to selection methods, most HIV-infected infants included in this analysis were relatively well, with a median CD4 count of 34%. The algorithm may perform better in a general population of HIV-infected infants. Additionally, only 12% of this cohort was breastfeeding. In conclusion, a combination of clinical findings in infants below 10 weeks of age may be useful for predicting HIV infection and prioritising care. There is urgent need to validate these findings in other settings and to determine their relevance in diverse settings.

## Conclusion

A combination of physical findings is helpful in identifying infants most likely to be HIV-infected. This may inform management algorithms and provide guidance for focused laboratory testing or antiretroviral initiation in some settings, and should be further validated in these settings and elsewhere.

## List of Abbreviations

HIV: Human immunodeficiency virus; CHER: Children with HIV Early Antiretroviral; WHO: World Health Organization; ART: Antiretroviral therapy; VTP: Vertical transmission prevention; DNA: Deoxyribonucleic acid; PCR: Polymerase chain reaction; NVP: Nevirapine; ZDV: Zidovudine; OR: Odds ratio; IQR: Interquartile range; ANOVA: Analysis of variance; LR: Likelihood ratio; IMCI: Integrated Management of Childhood Illness

## Competing interests

The authors declare that they have no competing interests.

## Authors' contributions

All authors read and approved the final manuscript. HBJ performed primary data analysis and was primary manuscript author; LM assisted with data analysis and manuscript development, and overall study design; SAM developed the cohorts and edited the manuscript; AV developed the cohorts and edited the manuscript; DMG provided scientific advice, clinical advice, and manuscript edits; WSS provided laboratory support and manuscript edits; ED contributed to clinical management of the cohort and was the primary clinical advisor; MFC provided the design and contributed to manuscript authoring.

## Pre-publication history

The pre-publication history for this paper can be accessed here:

http://www.biomedcentral.com/1471-2431/11/104/prepub
